# Analysis of axonal regeneration in the central and peripheral nervous systems of the NG2-deficient mouse

**DOI:** 10.1186/1471-2202-8-80

**Published:** 2007-09-27

**Authors:** Mohammed K Hossain-Ibrahim, Kia Rezajooi, William B Stallcup, Alexander R Lieberman, Patrick N Anderson

**Affiliations:** 1 Department of Anatomy and Developmental Biology, University College London, Gower Street, London WC1E 6BT, UK; 2 Department of Neurosurgery, Queen Elizabeth Hospital, Metchley Lane, Birmingham B15 2TH, UK; 3 The Burnham Institute for Medical Research, La Jolla, CA 92037, USA

## Abstract

**Background:**

The chondroitin sulphate proteoglycan NG2 blocks neurite outgrowth in vitro and has been proposed as a major inhibitor of axonal regeneration in the CNS. Although a substantial body of evidence underpins this hypothesis, it is challenged by recent findings including strong expression of NG2 in regenerating peripheral nerve.

**Results:**

We studied axonal regeneration in the PNS and CNS of genetically engineered mice that do not express NG2, and in sex and age matched wild-type controls. In the CNS, we used anterograde tracing with BDA to study corticospinal tract (CST) axons after spinal cord injury and transganglionic labelling with CT-HRP to trace ascending sensory dorsal column (DC) axons after DC lesions and a conditioning lesion of the sciatic nerve. Injury to these fibre tracts resulted in no difference between knockout and wild-type mice in the ability of CST axons or DC axons to enter or cross the lesion site. Similarly, after dorsal root injury (with conditioning lesion), most regenerating dorsal root axons failed to grow across the dorsal root entry zone in both transgenic and wild-type mice.

Following sciatic nerve injuries, functional recovery was assessed by analysis of the toe-spreading reflex and cutaneous sensitivity to Von Frey hairs. Anatomical correlates of regeneration were assessed by: retrograde labelling of regenerating dorsal root ganglion (DRG) cells with DiAsp; immunostaining with PGP 9.5 to visualise sensory reinnervation of plantar hindpaws; electron microscopic analysis of regenerating axons in tibial and digital nerves; and by silver-cholinesterase histochemical study of motor end plate reinnervation. We also examined functional and anatomical correlates of regeneration after injury of the facial nerve by assessing the time taken for whisker movements and corneal reflexes to recover and by retrograde labelling of regenerated axons with Fluorogold and DiAsp. None of the anatomical or functional analyses revealed significant differences between wild-type and knockout mice.

**Conclusion:**

These findings show that NG2 is unlikely to be a major inhibitor of axonal regeneration after injury to the CNS, and, further, that NG2 is unlikely to be necessary for regeneration or functional recovery following peripheral nerve injury.

## Background

NG2 is a large transmembrane proteoglycan of the chondroitin sulphate proteoglycan (CSPG) family, with a large ectodomain and a short cytoplasmic tail [1,2]. It is expressed in many different tissues, especially during development, but in the adult mammalian brain and spinal cord, it is expressed predominantly by a subset of glial cells with astrocyte-like morphology and the antigenic characteristics of oligodendrocyte progenitor cells [3]. These cells are present throughout white and grey matter at a density similar to that of oligodendrocytes and microglial cells, and in recent years it has been proposed that they constitute a novel class of glial cells (for which the term polydendrocytes has been proposed) with as yet poorly understood roles in adult nervous system function [3-5].

There is evidence from studies in vitro that NG2, like most other CSPGs, inhibits neurite outgrowth in culture [6], and possesses several domains that cause growth cone collapse [7]. It has also been shown that antibodies against NG2 block its inhibitory effects on neurite growth [8] and that among a variety of CSPGs expressed by a growth-inhibitory astrocyte cell line, NG2 is the one with by far the strongest inhibitory effects on neurite growth [9]. This evidence is complemented by evidence, from in vivo studies, that NG2 is present at sites at which regenerative growth of axons within or into the CNS is arrested, notably around CNS injury sites and at the dorsal root entry zone (DREZ), where NG2-expressing cells proliferate and accumulate after injury [10-15]; see reviews by Butt *et al*, 2002 [4], and Nishiyama, 2007 [16].

Findings such as these have led to a widespread belief that NG2 is one of the major inhibitors of axonal regeneration within or into the mammalian CNS after injury [17], a view that has been strongly reinforced by recent work reporting that neutralising monoclonal antibodies against NG2 applied at dorsal column lesion sites in adult rats, promote regeneration of sensory axons into the lesion site, and when combined with a conditioning lesion of the sciatic nerve, result in regenerative growth of axons rostral to the lesion site [18].

However, in addition to abundant evidence for the presence in the CNS of other molecules which are thought to inhibit regenerative axonal growth (including Nogo, myelin associated glycoprotein and oligodendrocyte myelin glycoproteins), there are numerous findings that cast doubt on the general validity of the view that NG2 is a major axon growth inhibitor and suggest the need for further investigation. For example, AN2, the mouse homologue of NG2, does not inhibit outgrowth of mouse dorsal root ganglion (DRG) cell neurites growing on a laminin substrate [19] and mouse cerebellar granule cells adhere to and extend neurites on substrates containing AN2 [20]. Also, whereas NG2 inhibits neurite outgrowth from rat cerebellar granule cells plated on a substrate of L1 (an axon-growth-promoting cell adhesion molecule), it has no effect on the outgrowth of neurites from DRG cells on the same substrate [6] suggesting that the inhibitory effects of NG2 are exerted selectively on only some types of neuron. There is also evidence that regenerating CNS axons may grow through regions rich in NG2-expressing cells [14,21]. Moreover, in very recent work, it has been shown that NG2-expressing cells promote neurite outgrowth from hippocampal and neocortical neurons in vitro and that both in vitro and in vivo NG2-expressing cells are preferentially and extensively contacted by axonal growth cones [22], in keeping with earlier evidence that NG2-expressing cells may receive synaptic input from glutamatergic axons [23,24].

The presence of NG2 in the normal and regenerating PNS raises further questions about its roles. NG2 is expressed by fibroblast-like cells and microvascular pericytes in peripheral [11,15,25], is present at nodes of Ranvier [15,26] and may also be expressed by a subset of non-myelinating Schwann cells [19]. Moreover, the peripheral axons of DRG cells in the sciatic nerve regenerate through the prominent cap of NG2-positive cells that forms over the proximal stump following resection of the nerve, and continue to grow along the distal stump in close proximity to NG2-expressing cells [15]. Such observations suggest that NG2 has no generalised inhibitory effect on regenerating PNS axons or that it is involved in subtle focal inhibitory functions directed at preventing profuse branching of the axons and/or perhaps serving to confine regenerating axons to the bands of von Büngner.

Our approach to elucidating the possible roles of NG2 in inhibiting axonal regeneration after injury was to compare regeneration and reinnervation in a variety of well-established CNS and PNS injury models, between normal and NG2 knockout mice. Thus we have studied axonal regeneration in the CNS after lesions of the corticospinal tract and dorsal column, and at the dorsal root-spinal cord interface following dorsal root injury. We have also examined regeneration and reinnervation in peripheral nervous system following facial and sciatic nerve injuries.

## Results

### Analysis of phenotype

**Figure 1:**
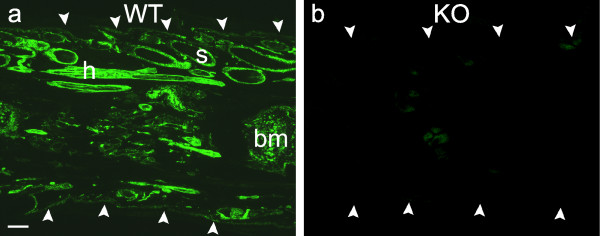
**Phenotyping of tailsnips**. Longitudinal sections of tail snips from a wild-type mouse (a) and a knockout mouse (b) immunoreacted with anti-NG2 antibody. Arrowheads delineate the edges of the tail snip sections. Strong NG2 immunofluorescence is apparent in hair follicles (h), sebaceous glands (s) and bone marrow (bm) and other structures in Fig. 1a but no NG2 fluorescence is detectable in Fig. 1b. Bar = 200 μm and also applies to Fig. 1b.

For all of the studies reported below, identification of knockout and wild-type mice was based on immunohistochemical phenotyping of tail snips with anti-NG2 antibody, which provided unequivocal evidence for the absence or presence of NG2 (see Fig. 1). No differences were apparent between knockout and wild-type mice with respect to appearance, behaviour, weight or gross features of brain and spinal cord.

### CNS injury models

#### Dorsal column injury

Following dorsal column lesions (and all other types of experimental injury) no differences were apparent at autopsy or in overall histological appearance of the lesion site or other areas of CNS tissue, between knockout and wild-type mice. In all cases the dorsal column above and below the lesion site was separated by a zone of collagenous connective tissue (scar tissue) which was adherent to the overlying meningeal tissue and was often lost during tissue processing, leaving a cyst-like cavity.

**Figure 2:**
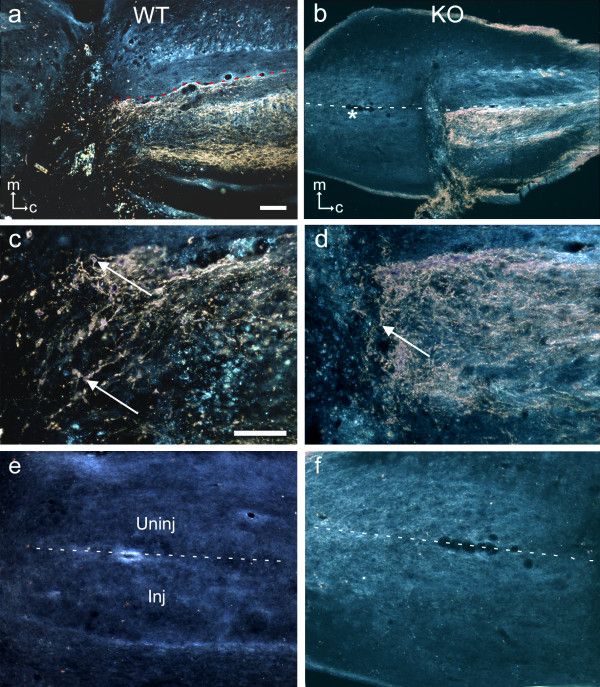
**Transganglionic labelling with CT-HRP of dorsal column axons after spinal cord injury**. Horizontal sections through the dorsal columns of the spinal cord of wild-type (Figs. 2a, c, e) and knockout (Figs. 2b, d, f) mice 28 days after unilateral transection of the dorsal column and ipsilateral sciatic nerve crush, and 3 days after injection of CT-HRP proximal to the sciatic nerve crush site. All sections were reacted for HRP. In Figs. 2a and b, at the lesion site (lesion marked with an asterisk and adjacent area enlarged in Figs. 2c and d) there is no indication of more extensive labelling or increased sprouting into or around the lesion site in the knockout relative to the wild-type control, and in both many axons displayed enlarged end bulbs (e.g. at arrows). Figs. 2e and f are taken approximately 2mm rostral to the lesion site. There are no regenerating or spared axons in the injured dorsal column in either. The dotted lines mark the midline; the injured dorsal column is below (to the left of) the midline in both figures. The orientation markers in Figs. 2a and b show medial (m) and caudal (c) for the left (injured) dorsal column. Scale bars = 100 μm; the bar in Fig. 2a applies to 2b also; the bar in Fig. 2c applies to 2d–f also.

In both knockout and wild-type mice surviving 28 days after transection of the dorsal column at T8 (and sciatic conditioning lesion), axons labelled transganglionically with CT-HRP were present in the dorsal column below the level of the lesion (Figs. 2a, b), but with the exception of a small number of axons which appeared to penetrate into the most caudal region of the lesion (Figs. 2c, d), labelled axons were not detected at more rostral levels (Figs. 2e, f). Furthermore, most of the labelled axons at the caudal margin of the lesion displayed enlarged end-bulbs suggesting arrested regeneration at this level. Systematic examination of serial sections showed no labelled axons growing around the lesion or bypassing it ventrally.

#### Dorsal root injury

**Figure 3:**
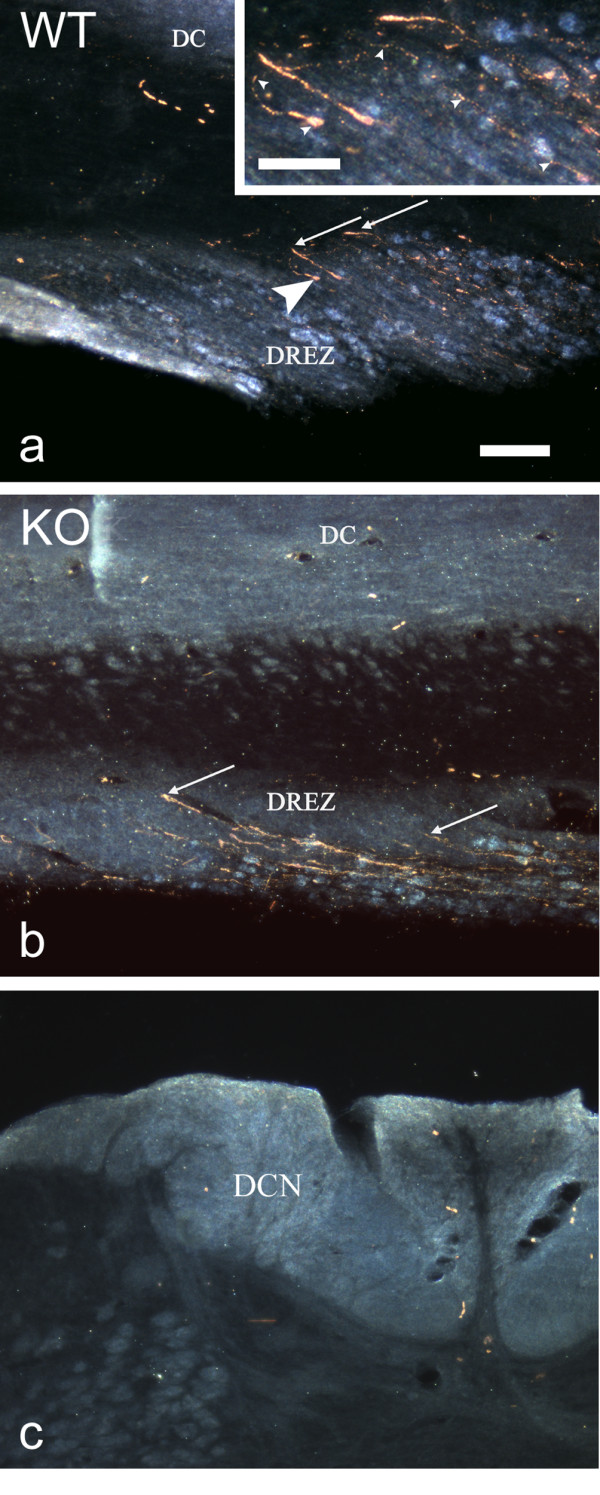
**Transganglionic labelling with CT-HRP of dorsal roots after transection and conditioning lesion**. The dorsal root entry zone (DREZ) of a wild-type (Fig. 3a) and knockout mouse (Fig. 3b) in horizontal sections and the dorsal column nucleus (DCN) in transverse section 28 days after transection and reapposition of the cut ends of the L5 dorsal root and crush of the ipsilateral sciatic nerve, and 3 days after injection of CT-HRP into the sciatic nerve proximal to the crush site. All sections were reacted for HRP. In both the wild-type and knockout almost all labelled axons (e.g. at arrows) stopped at the DREZ or turned back towards the dorsal root. The dorsal columns (DC) are devoid of labelled axons in both. The area indicated by the large arrowhead in Fig. 3a is enlarged in the inset, and illustrates a regenerating axon (indicated by small arrowheads) that turns back at the DREZ and displays a large terminal end bulb. Fig. 3c shows absence of labelled (spared) axons at the level of the DCN in a wild-type mouse. Scale bar = 100 μm and applies also to 3b and c. Inset scale bar = 50 μm.

In both knockout and wild-type mice 28 days after L5 dorsal root transection (and sciatic nerve conditioning lesion) labelled axons (transganglionic CT-HRP) were present in the dorsal root central to the injury site. However, the great majority appeared to be arrested at the DREZ where some formed end-bulbs (Figs. 3a, b) and some appeared to turn back into the root (Fig. 3a). No labelled axons were detected in the dorsal column or dorsal column nuclei (Fig. 3c) in either knockout or wild-type animals.

#### Corticospinal tract injury

**Figure 4:**
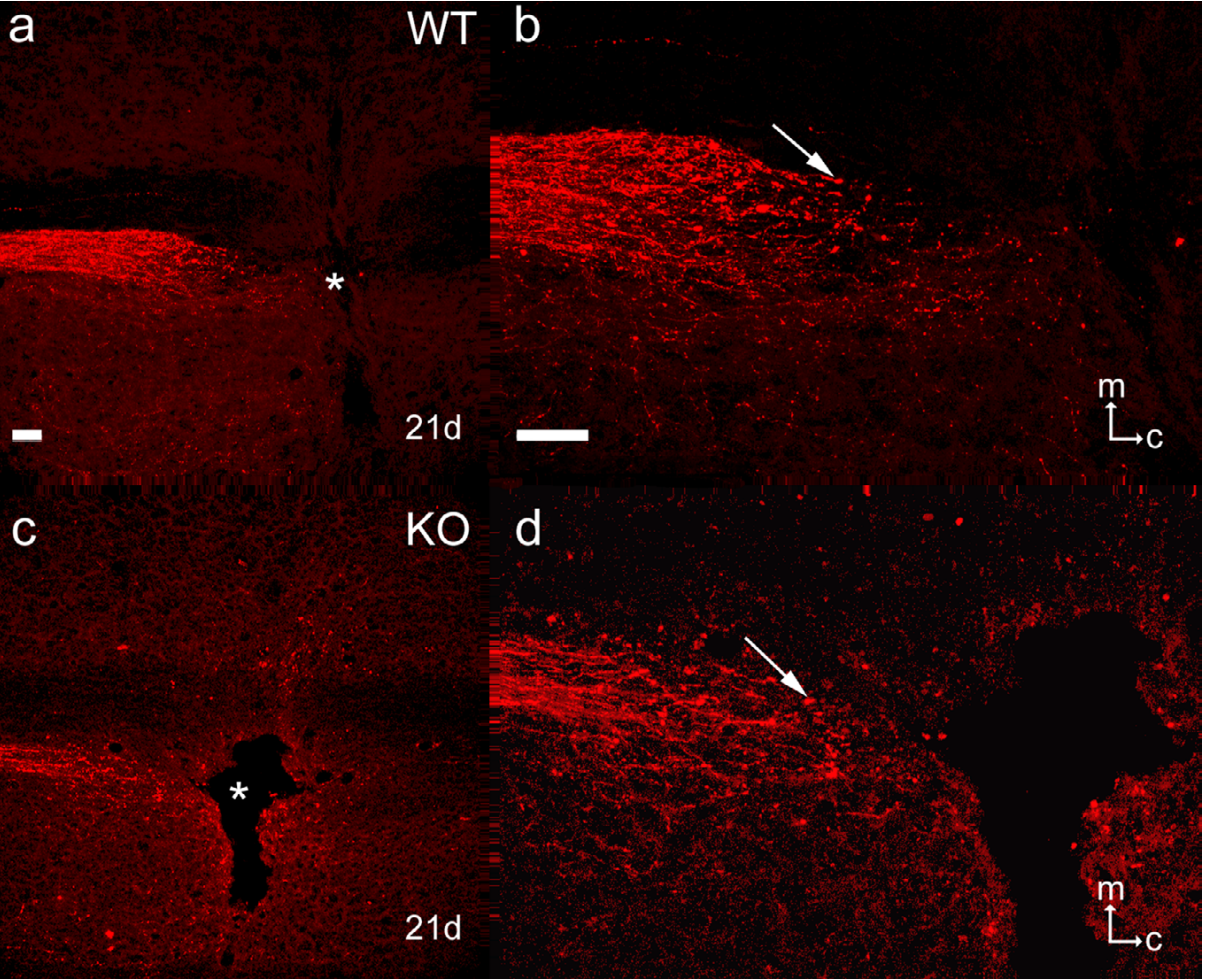
**Anterograde labelling with BDA of the CST after spinal cord injury**. Horizontal sections of the deep dorsal columns of low cervical spinal cord at and immediately rostral to the site of a unilateral transection of the dorsal column (including the CST) 21 days after the lesion and simultaneous anterograde labelling of the CST by application of BDA to the contralateral sensory-motor cortex, in a wild-type mouse (Fig. 4a, enlarged in Fig. 4b) and a knockout mouse (Fig. 4c, enlarged in Fig. 4d). There is no apparent enhancement of labelling in the knockout mouse relative to the wild-type, and in both examples CST axons terminate just rostral to the lesion cavity (asterisk in Figs. 4a and c), some with large end bulbs (e.g. arrows in Figs. 4b and d). The apparent labelling medial to the injury site in Fig. 4c is actually non-specific autofluorescence. The orientation markers on Figs. 4b and d show medial (m) and caudal (c). Scale bars on Figs. 4a and b = 100 μm and apply also to 4c and d.

Twenty-one days after transection of the dorsal column (incorporating the corticospinal tract, CST) at C6 and anterograde labelling of CST axons with BDA injected into the contralateral sensorimotor cortex, labelled axons were abundant in the CST rostral to the injury site but appeared to be arrested at the rostral border of the latter in both wild-type (Figs. 4a, b) and knockout mice (Figs. 4c, d). Many of the labelled axons displayed terminal end-bulbs in this area. No labelled axons penetrated the lesion site, none was observed bypassing the lesion site laterally or ventrally, none was found in the dorsal part of CST caudal to the lesion site, and there was no evidence to suggest that axons in the injured CST had grown or sent branches into the intact contralateral CST in either group. Fine lateral branches were observed to emerge from labelled CST axons rostral to the lesion site (not illustrated) but these were as likely to have been normal collateral branches as regenerative sprouts and no difference in the distribution or frequency of such side branches was detected between knockout and wild-type mice.

### Functional recovery after peripheral nerve injury

#### Sciatic nerve crush and transection

**Figure 5:**
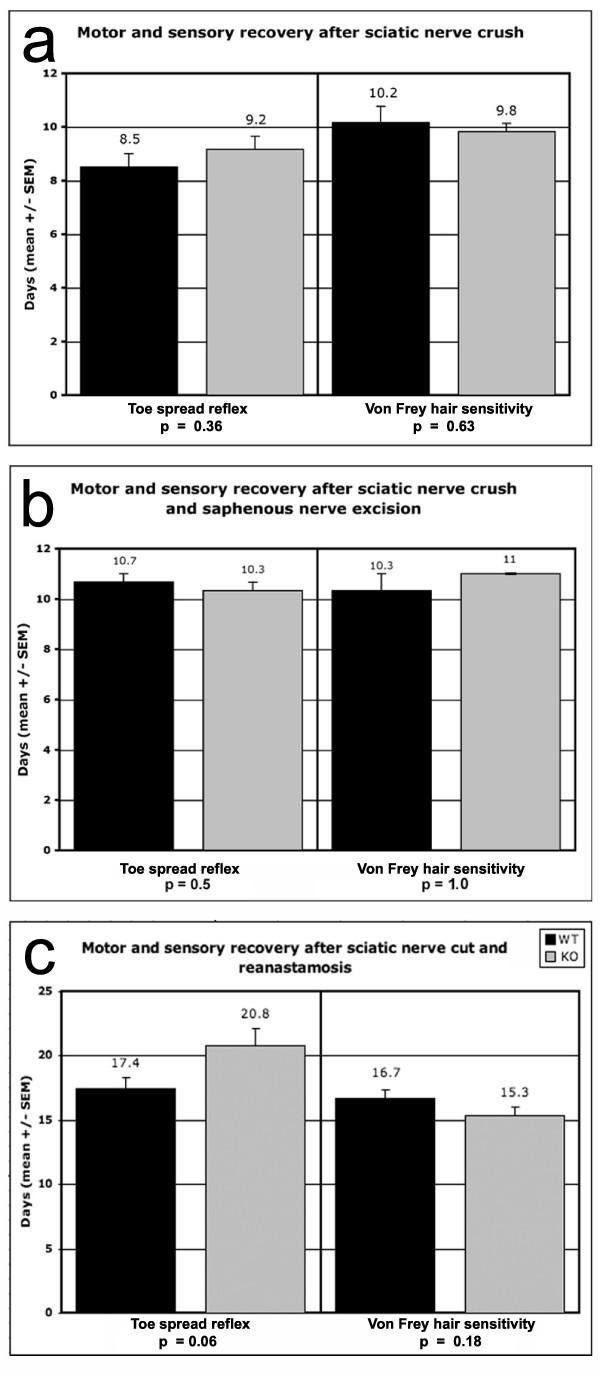
**Functional recovery after sciatic nerve injury**. Histograms illustrating the time taken for the recovery of motor function (assayed by toe spreading reflex) and sensory function (assayed by sensitivity to Von Frey hairs) after peripheral nerve injury in wild-type (black columns) versus knockout mice (grey columns). Group size was N = 6 wild-type and N = 6 knockout for Fig. 5a and b and N = 9 wild-type and N = 9 knockout for Fig. 5c. Standard error of the mean is indicated for each column. The differences between wild-type and knockout mice in time taken to recover sensory and motor function were not significant for any of the lesion groups (sciatic nerve crush, Fig. 5a; sciatic nerve crush and saphenous nerve excision, Fig. 5b; sciatic nerve transection and reanastamosis, Fig. 5c).

Recovery of sensory function, as a measure of the rate and extent of regeneration of sensory axons in the injured sciatic nerve, was assessed by twice-daily monitoring of the withdrawal reflex in response to stimulation of the lateral hindpaw with Von Frey hairs from 1 day following nerve injury to 2 days after full recovery was first observed. The threshold stimulus for eliciting withdrawal was identical for both hindpaws preoperatively and for the control hindpaw postoperatively in both wild-type and knockout mice. After sciatic nerve crush, wild-type mice recovered the preoperative withdrawal reflex threshold after a mean of 10.2 days and knockout mice after a mean of 9.8 days (p = 0.63 n.s.; Fig. 5a). After simultaneous excision of 3 mm of the saphenous nerve, recovery times were not significantly different: the withdrawal reflex was restored by 10.3 days in wild-type mice and by 11.0 days in knockout mice (p = 1.0, n.s. (calculated using a two tailed Fisher exact test because all 6 NG2 knockout mice recovered on the same day); Fig. 5b). These results confirm that recovery times after sciatic nerve injury alone could not be due to reinnervation as a result of sprouting from branches of the saphenous nerve, which, if it occurred, might give rise to false positive results. After sciatic nerve section and reanastamosis, which presents a greater barrier to regeneration than crush injury, recovery times were 16.7 days on average for wild-type and 15.3 days for knockout mice (p = 0.18 n.s.; Fig. 5c).

Recovery of motor function, as a measure of the rate and extent of regeneration of motor axons in the injured sciatic nerve, was assessed at the same time that sensory function was tested, by recording the toe spreading reflex when the hindpaw is raised above the supporting surface (this test is dependent on motor innervation of hindpaw muscles and is lost after sciatic nerve injury). The toe spreading reflex was restored, on average, at 8.5 days in wild-type and 9.2 days in knockout mice after sciatic nerve crush (p = 0.36 n.s.; Fig. 5a) and at 10.7 versus 10.3 days after simultaneous sciatic nerve crush and saphenous nerve resection (p = 0.50 n.s.; Fig. 5b). After sciatic nerve transection and reanastamosis, the toe-spreading reflex recovered on average after 17.4 days in the wild-type versus 20.8 days in the knockout mice, but this apparent 3-day lag in recovery of knockout mice was not statistically significant (p = 0.06; Fig. 5c).

#### Facial nerve crush

**Figure 6:**
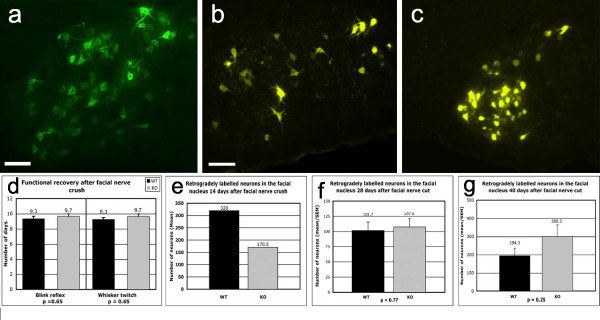
**Anatomical and functional correlates of regeneration after facial nerve injury**. Analyses of facial nerve regeneration in wild-type versus knockout mice after crush or transection of the facial nerve. Fig. 6a illustrates DiAsp-labelled neurons in the ipsilateral facial nucleus of a knockout mouse 14 days after facial nerve crush; scale bar = 100 μm. Fig. 6b shows Fluorogold (FG)-labelled neurons in the facial nucleus of a wild-type mouse in a coronal section of the ipsilateral pons 28 days after facial nerve transection; scale bar = 100 μm and applies to Fig. 6c also. Fig. 6c shows FG-labelled neurons in the contralateral (uninjured) facial nucleus of the same mouse as Fig. 6b. Note that some injured neurons are larger and that they are distributed throughout the facial nucleus rather than being clustered at its lateral edge, as in the uninjured facial nucleus. Fig. 6d shows histograms of the time taken for the blink reflex and spontaneous whisker twitching to reappear after facial nerve crush in 4 wild-type (black columns) versus 4 knockout (grey columns) mice. The means and standard errors of the means are shown at the top of each column. No differences were apparent between the wild-type and knockout groups for either test. Retrogradely labelled neurons were counted in the facial nucleus of 3 wild-type versus 2 knockout mice 14 days after facial nerve crush (DiAsp labelling; Fig. 6e – no statistical analysis performed), in 8 wild-type versus 8 knockout mice 30 days after facial nerve transection (FG labelling; Fig. 6f) and in 4 wild-type versus 4 knockout mice 40days after facial nerve transection (DiAsp labelling; Fig. 6g). Differences between wild-type and knockout mice do not reach significance, at any survival time and with either retrograde label.

Recovery of motor function after facial nerve crush was assessed twice daily, from 1 to 14 days postoperatively, by monitoring spontaneous whisker twitching and the corneal blink reflex. Whisker twitch took, on average, 9.3 days to recover in the 4 wild-type animals and 9.7 days in the 4 knockout animals (p = 0.65, n.s.; Mann-Whitney), with the corneal reflex recovering in 9.3 days versus 9.7 days (p = 0.65, n.s.; Mann-Whitney). Thus there was no significant difference between wild-type and knockout mice with respect to regeneration of facial nerve axons (Fig. 6d).

### Anatomical studies after peripheral nerve injury

#### Retrograde labelling of DRG cells

The number of DRG cells with axons that had regenerated into the glabrous skin of the hindpaw at various time points following sciatic nerve crush was determined by retrograde labelling with DiAsp to provide information on both the extent and timing of reinnervation. Di Asp was injected two days prior to sacrifice, because it takes this time to be taken up by the axons and retrogradely transported in sufficient quantity to fill the neuronal cell bodies. Because of variable uptake of DiAsp after injection into the hindpaw and variability in the intensity of neuronal cell body labelling, the threshold for identifying (and counting) labelled cells was set at a relatively high level. The numbers of labelled cells recorded may therefore represent gross underestimates of the actual number of DRG cells with regenerated axons. In all cases both feet were injected with DiAsp, and counts of labelled cells were made in both ipsilateral and contralateral L4 and L5 DRG as a control for inter-animal variability in uptake of DiAsp.

**Figure 7:**
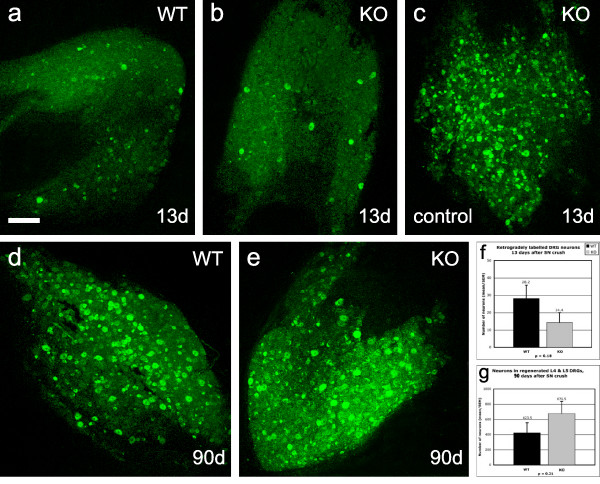
**Retrograde labelling of DRG after sciatic nerve injury**. Anatomical analyses of regeneration of sensory axons after sciatic nerve injuries in wild-type versus knockout mice. Figs. 7a-c show composite images made up by merging the entire z-series of optical sections through L5 DRG in wild-type (Fig. 7a) and knockout mice (Fig. 7b) 13 days after sciatic nerve crush and 2 days after injection of DiAsp into the skin of the hindpaw. Fig. 7c shows the control DRG (no sciatic nerve injury) from the same animal as Fig. 7b. Figs. 7d and e show a similar comparison of labelling in wild-type and knockout mice 90 days after sciatic nerve crush. Scale bar = 200 μm and applies to Figs. 7a-e. Figs. 7f and g compare the numbers of labelled cells in L5 DRG 13 days (Fig. 7f) and 90 days (Fig. 7g) after sciatic nerve crush in wild-type (N = 8 + 8; black columns) versus knockout mice (N = 8 + 8; grey columns). The quantitative analyses show that there are no statistically significant differences in the numbers of retrogradely labelled DRG cells (and thus in the extent of sensory axon regeneration) between wild-type and knockout mice at either short or long postoperative survival times.

At 5 and 9 days after sciatic nerve crush (2 animals at each survival time), the contralateral DRG were heavily labelled but there were no labelled cells in ipsilateral L4 or L5 DRG (data not shown) indicating that the crush lesions were complete, that L4 and L5 DRG are not labelled via routes other than injured sciatic nerve, and that regenerating sensory axons had not reached the hindpaw at these survival times. Very few labelled neurons were present in the ipsilateral DRG 13 days after sciatic nerve crush (i.e. 11 days after DiAsp injection into hindpaw), in either wild-type or knockout mice, with an average of 28 labelled neurons per DRG in wild-type mice compared to 14 in knockout mice (p = 0.18 n.s.; Figs. 7a, b and 7f). The appearance of retrograde label in injured DRG neurons 13 days after sciatic nerve crush, but not before, correlates with the onset of functional recovery being 9–10 days after sciatic nerve crush in our other experiments (see Figure 5a and 5b).

Much larger numbers of labelled DRG cells were present 90 days after injury, averaging 424 per DRG in the wild-type mice and 677 in the knockout mice (p = 0.27 n.s.; Figs. 7d, e and 7g). Thus, differences between wild-type and knockout mice groups were not significant at either 13 or 90 days after injury.

There were no significant differences in the numbers of retrogradely labelled DRG cells in the L4/5 DRG on the control side between wild-type and knockout mice at either 13 days (see Fig. 7c for example); on average 138 labelled neurons in the wild-type and 135 in the knockout; p = 0.96) or at 90 days (on average 489 labelled cells in the wild-type and 627 in the knockout mice; p = 0.21). The marked phenotype-independent difference in the number of labelled neurons in the contralateral (control) L4 and L5 DRG at 13 days versus 90 days was, however, a consistent and unexpected finding. A possible explanation for this may be that DiAsp is a more effective retrograde label for DRG cells in mature animals than in young ones.

#### Reinnervation of denervated endplates in the soleus muscle

**Figure 8:**
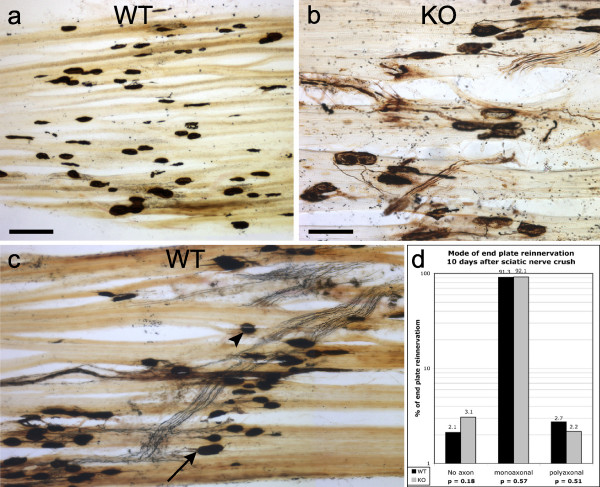
**Silver-cholinesterase stained motor end plates after sciatic nerve injury**. Longitudinal sections of soleus muscle 7 days (Fig. 8a) and 10 days (Figs. 8b and c) after sciatic nerve crush showing that regenerating axons have not reached/reinnervated end-plates at 7 days but extensive reinnervation has occurred by 10 days in both wild-type (Fig. 8c) and knockout (Fig. 8b) mice. Some endplates were apparently not reinnervated at 10 days (e.g. at arrowhead in Fig. 8c) and a few appeared to be innervated by more than one axon (e.g. at arrow in Fig. 8c). Bar = 100 μm in a and applies to c also; bar = 50 μm in b. Fig. 8d shows quantitative data on the extent of reinnervation of end-plates 10 days after sciatic nerve crush, expressed as the percentages of end-plates without axons, with a single reinnervating axon and with more than one reinnervating axon for wild-type (N = 6; black columns) and knockout mice (N = 6; grey columns). No significant differences in the timing, extent or type of reinnervation were detected.

The progress and extent of muscle reinnervation 7 and 10 days after sciatic nerve crush was examined by light microscopy, using silver-cholinesterase histochemistry. Endplates in control soleus muscle from both wild-type and knockout mice were almost all innervated by a single axon (data not shown). At 7 days after injury no endplates had been reinnervated in either group (Fig. 8a). At 10 days, however, there was extensive reinnervation of endplates in both wild-type (Fig. 8c) and knockout mice (Fig. 8b) and no consistent differences in the appearance of the endplates were observed between the two groups. The mean percentage of non-innervated endplates at 10 days was 2.1% in wild-type and 3.1% in knockout mice (p = 0.18 n.s.) and the proportion of endplates reinnervated by a single axon was 91.3% in wild-type and 92.1 % in knockout mice (p = 0.57 n.s.), the proportion of polyinnervated endplates (2.7% in wild-type and 2.2% in knockout mice) was not significantly different between the two groups (p = 0.51; Fig. 8d).

#### Sensory reinnervation of hindpaw glabrous skin

**Figure 9:**
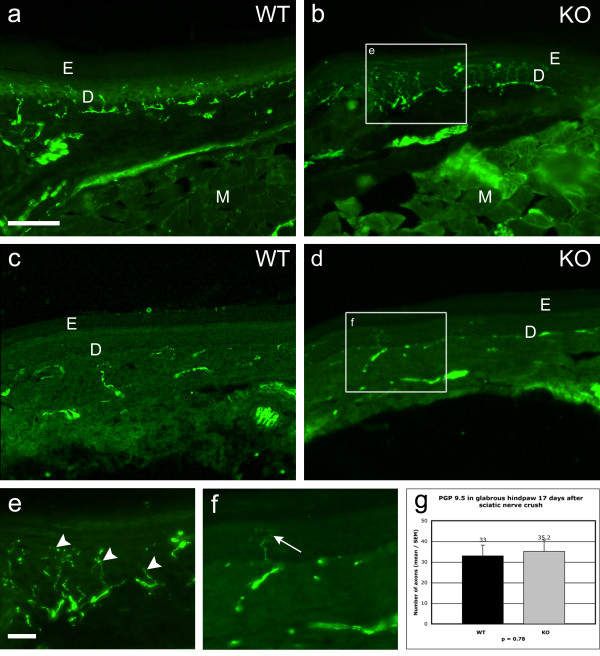
**PGP 9.5 immunohistochemistry for axons in plantar hindpaw skin after sciatic nerve injury**. Sections of glabrous hindpaw skin in wild-type (Figs. 9a, c and e) and knockout mice (Figs. 9 b and d) illustrating the extent of sensory reinnervation (PGP 9.5 immunopositive axons) after sciatic nerve crush. Figs. 9a and b show sections from uninjured wild-type and knockout mice, and Figs. 9c and d show similar areas of wild-type and knockout mouse skin 17 days after sciatic nerve crush. In the control material the sensory axons are concentrated in the superficial dermis (D) but are not present in the epidermis (E) or in subdermal musculature (M) and their distribution and extent is comparable in both wild-type and knockout mice. Seventeen days after nerve crush a few sensory axons have regenerated into the dermis to about the same extent in both wild-type and knockout animals. Figs. 9e and f are enlargements of the boxed areas in Figs. 9b and d, and illustrate the relative paucity of regenerating PGP 9.5+ axons in the dermis 17 days after sciatic nerve section (e.g. at arrow in Fig. 9f) compared with the dense innervation in control skin (e.g. at arrowheads in Fig. 9e). Bar in Fig. 9a = 100 μm and also applies to b – d; bar in Fig. 9e = 20 μm and applies to f also. Fig. 9g compares counts of PGP 9.5-positive axons in glabrous hindpaw skin 17 days after sciatic nerve crush, in wild-type (N = 6) versus knockout mice (N = 6). There is no significant difference.

The extent of sensory reinnervation of the glabrous skin of the hindpaw was assessed at 17 days after sciatic nerve crush by light microscopy after immunostaining for PGP 9.5. No differences were observed between wild-type and knockout animals on the control or injured side (Figs. 9a–f). In the sampled microscope fields (see Methods) the mean number of immunostained (presumptive regenerated) sensory axons in the dermis was 33 for the wild-type group and 35 for the knockout animals (p = 0.78 n.s.; Fig. 9g). There was an apparent difference between the wild-type and knockout animals with respect to PGP 9.5-positive nerve fibres in the uninjured hindpaw where a mean of 120 axons was counted in the wild-type versus 72 in the knockout, but this finding was based on only 3 animals per group, and significance could not be reliably established (p = 0.1, Mann-Whitney test).

#### Axon counts in digital nerves after sciatic nerve crush

**Figure 10:**
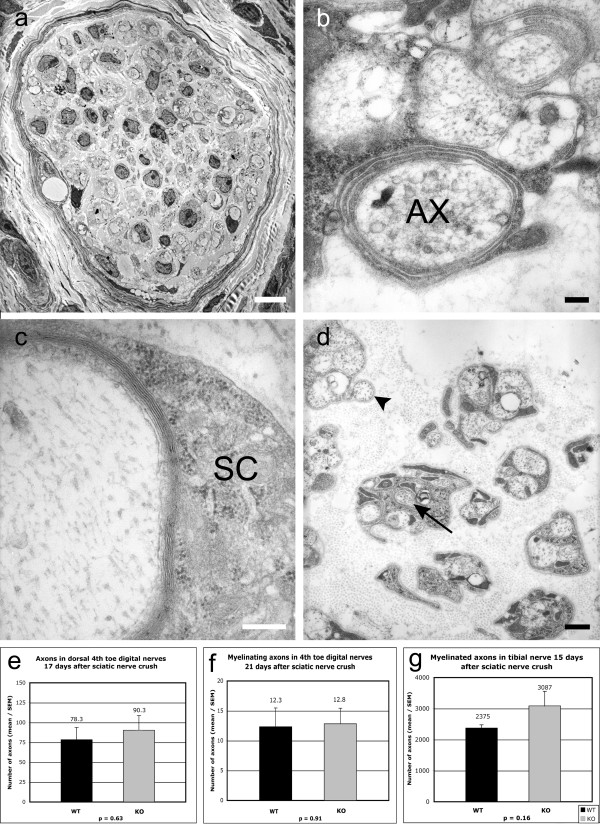
**Electron microscopy of regenerating axons in the digital and tibial nerves**. Figs. 10a – d shows transverse sections of digital nerves of the fourth toe 21 days after sciatic nerve crush. In Fig. 10a an entire nerve is shown at low magnification (bar = 10 μm). Fig. 10b shows a regenerating axon (AX) with 2–3 wrappings of Schwann cell processes ('premyelinated' axon; bar = 200 nm), and Fig. 10c shows a large regenerating axon with a thin semi-compacted myelin sheath and satellite Schwann cell (SC) (remyelinating axon; bar = 200 nm). Both of these axons would have been counted in the analysis for Fig. 10f. Fig. 10d shows unmyelinated regenerating axons, some surrounded by a thin layer of Schwann cell cytoplasm (at arrowhead) and others embedded within Schwann cell cytoplasm (e.g. at arrow), neither of which would have been counted. Bar = 500nm. Figs. 10e shows counts of all axons and Fig. 10f of remyelinating axons only in the digital nerves of the fourth toe of wild-type (N = 6; black columns) and knockout mice (N = 6; grey columns) 17 days (Fig. 10e) and 21 days (Fig. 10f) after sciatic nerve crush. Difference between axon counts in wild-type and knockout mice are insignificant. Fig. 10g shows counts of myelinated and remyelinating axons in the tibial nerve 15 days after, and about 5 mm distal to, a crush lesion of the sciatic nerve in 8 wild-type (black column) and 6 knockout mice (grey column). Although the mean fibre count was 3087 in the knockout and 2375 in the wild-type mice, this difference was not statistically significant.

Ultrathin sections of the digital nerves in the fourth toe distal to a sciatic nerve crush were analysed by electron microscopy 17 and 21 days after a sciatic nerve crush to compare numbers of regenerating unmyelinated and myelinated axons (e.g. Fig. 10a). There were no gross morphological differences in the ultrastructure of the digital nerves of wild-type and knockout mice 17 days after a sciatic nerve crush. All axons were unmyelinated and the mean number counted in the dorsal digital nerves of wild-type mice was 78.3 (median 75.5; SD 38) compared to a mean of 90.3 axons in the knockout mice (median 93; SD 45) (p = 0.63; Fig. 10e). In the mice surviving 21 days after sciatic nerve crush, premyelinating axons (Fig. 10b) and remyelinating axons (Fig. 10c) were counted in all digital nerves (Fig. 10a) of the ipsilateral fourth toe. Wild-type mice had a mean of 12.3 (partially) myelinated axons in the 4^th ^toe digital nerve (median 11.5; SD 7.71) compared to a mean of 12.8 axons (median 12.5; SD 6.37) in the knockout mice (p = 0.91; Fig. 10f). In summary, the regenerative growth of sensory axons into digital nerves after sciatic nerve crush appears to be unaffected by the NG2 null mutation.

#### Axon counts in the tibial nerve after sciatic nerve crush

Transverse sections of the tibial nerve 5 mm distal to a sciatic nerve crush injury site were analysed 15 days after injury. The mean number of myelinated axons in wild-type mice was 2375, compared to 3087 in knockout mice. Estimates of the surface area of the wild-type and knockout tibial nerve cross sections were sufficiently similar to suggest that overall size differences in the nerve cross sections were unlikely to be a confounding factor in the numerical estimates. The difference in the number of regenerating myelinated axons between wild-type and knockout animals was not statistically significant (p = 0.164; Fig. 10g).

#### Facial motor nucleus neuron counts after facial nerve injury

The numbers of facial nucleus neurons retrogradely labelled with Fluorogold (FG) applied to the whisker pad 27 days after facial nerve transection and 3 days before the animals were killed, were determined in 8 wild-type and 8 knockout mice (Fig. 6b). The mean number of labelled neurons in the wild-type mice was 101.7 and in knockout mice was 107.6 (p = 0.77 n.s.; Student's t-test; Fig. 6f).

In order to confirm that retrograde label was being uptaken sufficiently, a further 4 wild-type and 4 knockout mice received DiAsp 37 days after facial nerve transection (3 days before sacrifice). Wild-type mice had a mean of 194.3 labelled neurons and the knockout mice a mean of 300.3 labelled neurons (p = 0.25 n.s.; Mann-Whitney test; Fig. 6g).

Similarly, the numbers of facial nucleus neurons retrogradely labelled with DiAsp applied to the whisker pad 12 days after facial nerve *crush *(at the same level as transection injury) and 2 days before the animals were killed, were determined in three of the wild-type and three of the knockout mice used for assessing functional recovery (one of which was excluded because of poor retrograde labelling; Fig. 6a). The mean number of labelled neurons in wild-type mice was 320 and in knockout mice was 170.5. This difference could not be tested for statistical significance because of the small number of animals included in these groups (Fig. 6e).

The fact that there were apparently more DiAsp-labelled neurons in the knockout mice than in the wild-type mice after facial nerve transection, but more in the wild-type than in the knockout mice in the facial nerve crush group further suggests that the difference between wild-type and knockout animals in the crush group was not significant and reinforces the conclusion that the absence of NG2 in the knockout mice has no detectable effect on the regeneration of axotomised facial nerve axons.

These findings are entirely consistent with those obtained with FG, thus strengthening confidence in DiAsp-based results. The control side facial nucleus had a mean of 305.4 neurons labelled with FG in the wild-type mice and 279 neurons labelled in the knockout mice (p = 0.3 n.s.; Student's t-test; Fig. 6c), suggesting that there was very little variability in FG uptake or retrograde transport of FG (data not shown).

## Discussion

### The major findings

The role of NG2 in the injured nervous system is controversial. Although NG2, a molecule widely regarded as a potent inhibitor of axonal regeneration, is strongly expressed at injury sites in the CNS, where regeneration is abortive, it is also strongly expressed in injured peripheral nerves, where axonal regeneration is vigorous (e.g. [10,11,15,27]; and see review by [4]). It might have been expected, therefore, that the absence of NG2 would enhance axonal regeneration in the CNS and/or reduce regeneration in peripheral nerves. Our findings were consistent and in some respects surprising: the absence of NG2 had no effect on axonal regeneration in vivo. Specifically, we could find no evidence that regenerative growth of dorsal root axons into the spinal cord after dorsal root injury, or of ascending (sensory) axons within the spinal cord following dorsal column injury, was enhanced in the NG2 knockout mice. Similarly, descending corticospinal tract axons failed to show enhanced regeneration in NG2 knockout mice following spinal cord injury. In addition, we have shown, by quantitative methods and for the first time, that sciatic nerve fibres, both motor and sensory, and facial (motor) nerve fibres, regenerated and re-established functional innervation of target tissues to the same extent and at the same speed in the NG2 knockout and wild-type groups.

Because there is evidence that neurons may vary in their sensitivity to NG2 (see below) it is important that we have examined regeneration in a number of models. Two different populations of projection axons within the spinal cord (dorsal column sensory axons and CST axons), dorsal root axons at the PNS/CNS interface, and motor and sensory axons of two peripheral nerves (sciatic and facial) were all tested for possible effects of the absence of NG2 on regeneration after axotomy. Furthermore, in the case of the injured peripheral nerve, different types of lesion were examined, and both functional and multiple types of anatomical analyses of regeneration were carried out. In all of the systems and experimental conditions tested we found only insignificant differences between NG2-deficient and normal animals. We therefore conclude that: i) NG2, on its own, does not exert a profound influence on the regenerative growth of peripheral nerve fibres; and ii) absence of NG2 from the microenvironment of the CNS does not enhance the regenerative growth of injured axons or facilitate the entry of regenerating dorsal root axons into the CNS even when the regenerative potential of the DRG cells is enhanced by a conditioning lesion. In other words, NG2 cannot be viewed as a major cause of failure of axonal regeneration within or into the CNS. The latter conclusion is entirely in keeping with the recent findings of de Castro *et al*. [28], who carried out closely related studies on NG2 knockout mice. They transected the spinal cord at vertebral level T9/10 and found that the penetration of immunohistochemically visualised CGRP-containing (presumptive sensory axons) into the NG2-rich scar tissue at the transection site was no more extensive in the knockout mice than in the wild-type controls, and that penetration into the scar tissue of serotoninergic (5HT-immunopositive) axons was *greater *in the wild-type than in the knockout mice.

Our findings and conclusions are also in line with other evidence that NG2 is neither a major inhibitor of axonal regeneration in the CNS nor a promoter of longitudinal regeneration in peripheral nerves. This includes the recent work of Yang *et al*. [22] which provides some of the strongest and most direct in vitro evidence that NG2-expressing cells do not inhibit or repel growing/regenerating neurites, and indeed provides both in vitro and in vivo data suggesting the opposite, albeit based heavily on CNS neurons derived from neonatal rather than mature animals (see below).

### NG2 as an inhibitor of axonal regeneration in the CNS

A number of previous in vivo studies have suggested that NG2 and other CSPGs play an important part in limiting axonal regeneration in the CNS. NG2 is the most strongly upregulated CSPG at CNS injury sites, with a time course very closely matched to the arrested growth of axonal sprouts [13,27], and enzymatic degradation of CSPGs (in particular, removal of chondroitin sulphate glycosaminoglycans by chondroitinase ABC) promotes regenerative axonal growth in the adult CNS [29-31]. However, such enzymatic degradation is not specific to NG2 and will affect many other molecules with potential axon growth inhibiting properties and it is relevant that there are strongly inhibitory domains in the NG2 core protein which would be unaffected by treatment with chondroitinase ABC [7]. More recently, Tan et al., [18] have described more direct evidence that NG2 is involved in inhibiting CNS regeneration by well controlled studies of regenerative growth of transganglionically CTB-labelled dorsal column axons at thoracic cord transection lesion sites in young adult rats. They demonstrated a convincing enhancement of growth into (and beyond) the lesion site, when NG2 was neutralised with function-blocking monoclonal antibodies applied directly at the lesion site, especially when the DRG neurons were primed with a conditioning lesion of the sciatic nerve. We recognise that the work of Tan *et al*. provides a strong challenge to the conclusion de Castro et al. and we have reached on the basis of our studies of NG2 knockout mice. A possible explanation of the effects of the antibody reported in the study of Tan *et al*. is that they may have resulted from modulation of the behaviour of NG2-expressing cells or cell complexes, rather than by direct blocking of axon/NG2 interactions.

### NG2 as a facilitator of regeneration in peripheral nerves

Whether or not NG2 has an insignificant role in preventing axonal regeneration in the CNS – as our findings suggest – or is a contributory factor in this process, NG2 is upregulated in vigorously regenerating peripheral. Hence it may be that NG2 has facilitatory roles with respect to axonal regeneration, especially in the PNS where it has been suggested that NG2 may promote enhanced mobility of non-neuronal cells after injury, thereby aiding the formation of cellular (Schwann cell) bridges to act as a supportive substrate for regenerating axons [15,32,33]. We have also previously suggested, based on its distribution within normal and injured peripheral nerve, that a plausible role for NG2 might be to facilitate regeneration by helping to confine regenerating axons within the endoneurial compartment and/or within the bands of Von Büngner of the distal stump [15]. However, our finding that regeneration of sensory and motor axons in peripheral nerves is not reduced or delayed in NG2 knockout mice suggests that any such effects are of very minor significance for peripheral nerve regeneration.

### Potential confounding factors

It is pertinent to consider why the absence from the transgenic mice of NG2, a molecule with powerful effects in vitro, had so little effect on axonal regeneration in vivo. First, it is possible that NG2 has a redundant role in this process since NG2 is only one of a number of potential growth inhibitory or repulsive molecules at injury sites in the nervous system. For example, NG2, tenascin-C and CSPGs other than NG2 are expressed in similar regions of lesion sites in spinal cord (e.g. the meningeal scar) and peripheral nerves (the perineurium and the surface of bands of Von Büngner) [27,34-37]. It would be interesting to analyse with immunohistochemistry whether these molecules are upregulated in the NG2 KO mice compared to wild-type mice in the tissues studied.

Second, elimination of the NG2 gene may have induced upregulation of other molecules with similar functions during development, thus further compensating for any effects of the absence of NG2 in the knockout animals. Conditional knockout and/or knockdown experiments (e.g. with siRNA) would be necessary to test this hypothesis.

Third, it is also possible that the neuronal populations investigated in this study were particularly insensitive to NG2. It is known that neuronal populations differ in their responsiveness to NG2 in vitro [6,19,20] and in vivo [28], and it is conceivable that (some of) the neuronal populations we examined do not normally express receptors for NG2 and are thus intrinsically less responsive to NG2 than other populations. However, among neuronal types that have been reported to show inhibition of neurite growth in the presence of NG2 are DRG cells [6,9] and neocortical neurons (albeit not specifically CST neurons [9]). This and the fact that we tested several types of neurons make it extremely unlikely that our findings are not generally applicable. It is also possible that age is a relevant factor when considering the negative findings in our study. Thus we used adult animals and studied regeneration in vivo whereas much of the data suggesting inhibitory effects of NG2 on axonal growth derive from in vitro studies using embryonic or neonatal neurons: for example, embryonic or neonatal rat (or chick) DRG neurons [6,9,38]; rat E17 cortical neurons [9]; E24-25 chick retinal ganglion cells [39]; and early postnatal rat cerebellar granule cells [6,8]. Inhibition by NG2 of regenerative axonal growth from adult neurons has not been directly demonstrated, and it would not be surprising if developing neurons differed significantly from their adult counterparts with respect to their responsiveness to NG2. However, in view of the body of (largely indirect) evidence favouring a role for NG2 in inhibiting axonal regeneration in the adult CNS (see Introduction and further discussion below), our failure to find evidence for such a role of NG2 is unlikely to be explicable solely in terms of the age/maturity of our experimental animals.

Experiments on mice lacking the putative myelin-derived inhibitory molecule Nogo-A have also failed to show consistent enhancement of axonal regeneration after injury [40-43], even though treatment with Nogo-A-blocking antibodies increase sprouting and regeneration of injured corticospinal neurons [44,45]. Similarly, genetically inactivating the Nogo receptor (NgR) failed to produce significant regeneration of one class of axon (the corticospinal tracts) that express the receptor strongly [46,47], whereas subcutaneous administration of the NgR antagonist NEP1-40 enhanced regeneration of corticospinal axons [48]. The reason for the discrepancy between genetic and pharmacological inactivation of both Nogo and NgR is not clear [49]. Part of the lack of effect of knocking out NG2 may have been due to the genetic background of the mice we studied- C57BL/6. Genetic background can have considerable effects on axonal regeneration in strains of transgenic mice. Recently Dimou et al. [50] showed that corticospinal axons regenerated less well in Nogo-A knockout mice with a C57BL/6 background (as used in the present study) than in knockout mice with a 129X1/SvJ background, a finding that may be produced by a weaker intrinsic regenerative response by neurons from the C57BL/6 strain. None the less, C57BL/6 mice can regenerate axons vigorously following peripheral nerve injury and intrinsic CNS neurons from such animals can regenerate axons into peripheral nerve implants in the brain (unpublished observations). If NG2 was the major factor preventing axonal regeneration in the spinal cord of C57BL/6 mice, some enhancement of regeneration in the knockout animals would have been expected.

## Conclusion

Our studies suggest that NG2 is not a major inhibitory molecule preventing regeneration in the CNS and, further, that it is not essential for successful axonal pathfinding, regeneration and functional reinnervation in injured peripheral nerves. It is conceivable that if CNS regeneration-promoting interventions such as enhancing the intrinsic regenerative ability of CNS neurons, expressing neurotrophic molecules to support regenerating axons and neutralising other putative inhibitory molecules were to be applied, NG2 knockout mice would show enhanced regeneration relative to wild-type mice. However, such studies have not yet been carried out and for the moment the evidence that NG2 is not a key inhibitor of axon regeneration appears to be very strong.

## Methods

### Phenotyping

NG2 knockout mice were bred with C57BL/6 wild-type mice (Harlan, UK) to produce a breeding stock of heterozygous mice, the offspring of which were phenotyped as follows. Tailsnips from all mice were sectioned at 12 μm in a cryostat and mounted on gelatinised glass slides along with sections of tailsnips from known wild-type mice as a positive control. The sections were post-fixed for 5 minutes with 4% paraformaldehyde in 0.1 M PBS (PFA), rinsed (x3) in 0.1 M TBS containing 0.05% Tween detergent (TNT), and incubated with 0.3% H_2_O_2 _for 15 minutes, rinsed again in TNT and placed for 1 hour in 10% normal goat serum, 1% BSA and 0.1% Triton X-100 (Sigma, Dorset, UK) made up in TBS. The polyclonal anti-NG2 antibody was then added (1:5000) and the sections incubated for 72 hours at 4°C. (A polyclonal antibody was used in preference to a monoclonal antibody because it would be expected to recognise many sites on the NG2 protein, and therefore be more likely to detect a (possibly functional) truncated form of NG2 in the KO mouse). The sections were then washed again in TNT (x3) and incubated in Alexafluor-488- conjugated goat anti-rabbit biotinylated IgG secondary antibody (Molecular Probes; 1:400) for two hours, washed in TBS and coverslipped with DABCO anti-fade medium. Mice with no evidence of NG2 staining, following comparisons with positive control tissue (see Fig. 1), were designated as knockouts (KO) and used in experiments with age and sex matched C57BL/6 mice as wild-type (WT) controls. Homozygous mutants were viable, fertile, and morphologically indistinguishable from wild-type mice.

### General surgical procedures

All animal procedures were performed in accordance with local ethical committee and UK Home Office approvals. Male and female adult mice, between 6 and 9 weeks old were used, with wild-type and transgenic mice matched for age and sex. For all experiments the animals were coded and randomised so that their phenotype was unknown at the time of surgery and in the course of data analysis. All surgical procedures were performed under aseptic technique and general anaesthesia. Animals were initially anaesthetised with 4% halothane and anaesthesia was maintained with 1.5% halothane (plus oxygen and nitrous oxide 1:1). In the case of the experiment in which DiAsp (Molecular Probes, Oregon, USA) was introduced into whisker pads, anaesthesia was induced by a single intraperitoneal injection of 150 μl of 2.5% Avertin (tribromoethanol; Sigma, Deisenhofen, Germany) per 10 g of body mass. In animals with sciatic nerve or dorsal root injuries, approximately 0.5 ml sucrose octa-acetate and denatonium benzoate (Mentholatum, Glasgow, UK) was applied to the hindpaw on the injured side to prevent autophagy. Following surgery, all animals received intramuscular buprenorphine (20 mg/kg) opiate analgesic (Vetergesic; Alstoe Ltd., Yorks., UK) and recovered in an incubator. See Table 1 for a summary of utilisation of animals.

**Table 1:** Utilisation of Animals

	**Number of mice**			
			
**Experimental procedure(s)**	KO	WT	**Survival period**	**Analysis performed**
Transection of DC at T8; conditioning lesion of ipsilateral sciatic nerve; subsequent injection of CT-HRP into sciatic nerve for TGL	8	6	21d	Regen growth by analysis of labelled sensory axons at/beyond DC lesion site
Transection of dorsal spinal cord at C6; simultaneous injection of BDA into contralateral sensorimotor cortex	6	6	21d	Regen growth by analysis of labelled CST axons at DC lesion site
Transection of L5 dorsal root; conditioning lesion of ipsilateral sciatic nerve; subsequent injection of CT-HRP into sciatic nerve for TGL	6	6	21d	Regen growth by analysis of labelled sensory axons in dorsal root and DREZ
Sciatic nerve crush Sciatic nerve crush; saphenous nerve resection Sciatic nerve transection	6 6 9	6 6 9	n/a n/a n/a	Recovery of foot withdrawal (sensory) and toe spreading (motor) reflexes
Sciatic nerve crush; subsequent injection of DiAsp into hindpaw skin	10	10	5d (n = 2) 9d (n = 2) 13d (n = 8) 90d (n = 8)	Extent of sensory axon regen by analysis of retrogradely labelled DRG cells
Sciatic nerve crush	9	12	7d (n = 6) 10d (n = 15)	Extent of motor regen by analysis of silver-cholinesterase preparations of soleus muscle
Sciatic nerve crush	6 6	6 6	17d 21d (EM only)	Sensory reinnervation of skin by PGP 9.5 IHC; regen of axons into digital nerves by EM
Sciatic nerve crush	6	8	15d	Regen of axons into the distal nerve stump by EM
Facial nerve crush: subsequent application of DiAsp to whisker pad	4	4	14d	Extent of axon regen by analysis of retrogradely labelled facial neurons (n = 8) & recovery of whisker twitch and blink reflexes (n = 5)
Facial nerve transection: subsequent application of FG or DiAsp to whisker pad	12	12	30d (FG, n = 8) 40d (DiAsp, n = 4)	Extent of axon regen by analysis of retrogradely labelled facial neurons

KO = knockout; WT = wild-type; d = days post op; TGL = transganglionic labelling; CST = corticospinal tract; DC = dorsal column; DREZ = dorsal root entry zone; IHC = immunohistochemistry; EM = electron microscopy; FG = Fluorogold; Regen = regenerative/regeneration.

### CNS injury models

#### Dorsal column and sciatic nerve conditioning lesions

A midline incision was made over the lower thoracic vertebrae, muscles overlying the spine were stripped and a bilateral laminectomy performed at T8 to expose the spinal cord. The dura was opened and two puncture holes were made with a fine insulin syringe, one immediately medial to the left dorsal roots and the second on the immediate opposite side of the midline. The points of a pair of straight-bladed microscissors were inserted vertically into the needle holes and the scissors closed to transect the left dorsal column. The insulin needle, marked at 2 mm, was passed across the injury site to ensure a uniform depth of lesion. Superficial muscles were sutured and the wound closed. To maximise the regenerative response of injured ascending axons in the dorsal column, a conditioning peripheral nerve injury was performed at the same time as the dorsal column lesion. The ipsilateral sciatic nerve was exposed at mid-thigh level, crushed for ten seconds with watchmakers' forceps (No. 7) and the skin incision was closed with Histoacryl glue (B. Braun, Melsungen AG, Germany). After 25 days, the animals were reanaesthetised, the left sciatic nerve exposed and an incision made into the nerve proximal to the previous crush site. A fine glass micropipette attached to a Hamilton syringe was pushed into the nerve through the incision, and 1 μl of CT-HRP (dissolved at 14 mg/ml; List Biological Laboratories) was slowly injected into the endoneurium. The micropipette was withdrawn and the sciatic nerve was ligated immediately above the incision in the nerve to prevent leakage of injectate. The skin incision was then sutured and the animals allowed to recover and survive for 3 further days.

#### Dorsal root injury

The lumbar spine was exposed via a midline dorsal incision and a unilateral left L4 and L5 laminectomy performed to expose the lower lumbar dorsal roots. The L5 root was identified, transected with microscissors and the cut ends reapposed and held together with fibrinogen (Sigma, Dorset, UK). The ipsilateral sciatic nerve was crushed as described above. After 25 days, the animals were reanaesthetised and 1 μl of CT-HRP was injected into the sciatic nerve as described above, in order to label axons in the dorsal root. As the purpose of these experiments was to test for the ability of regenerating axons to enter the CNS they are included under the general heading of CNS injury models.

#### Controls for spared axons

Because incomplete dorsal column (or dorsal root) lesions might result in CT-HRP labelling of uninjured axons in the dorsal column, transverse sections were cut through the medulla at the level of the gracile nucleus in every animal in these two experimental groups. These sections were processed alongside the sections of the injury site. Three animals with evidence of axon sparing (labelled axons in or immediately caudal to the gracile nucleus) were excluded from the study on the basis of these controls.

#### Corticospinal tract injury and labelling

The skin over the back of the neck was incised, muscles of the neck reflected, and the spinal cord exposed by bilateral laminectomy of vertebrae C6 and C7. The area of dorsal column between the DREZ and the midline on the left side was cut using a pair of microscissors, and the lesion was extended across the midline and checked to extend to a depth of 2 mm below the dorsal surface of the cord with a marked needle. Care was taken to undercut and avoid damage to the dorsal spinal vein. The dura was closed and overlying muscle and skin sutured. While the animals remained under anaesthesia, BDA (Molecular Probes, Oregon, USA; 10% solution in dissolved in 0.1 M PBS) was injected into the contralateral sensorimotor cortex to anterogradely label corticospinal tract axons. A 2 × 1 mm burr hole was made over the right parietal cortex, the dura opened, and a glass micropipette attached to a Hamilton syringe was pushed into the cortex from the rostral edge of the burr hole, at a very shallow angle and advanced for 2 mm in a caudal direction. The pipette tip was kept almost parallel to the cortical surface and at a depth of no more than 1 mm. BDA solution (1.7 μl) was then injected while the pipette was slowly retracted to its entry point. The pipette was then advanced through the same entry point, for the same distance and at the same depth but directed slightly medially with respect to the first injection and a second aliquot of BDA (1.7 μl) injected in the same manner as the first. Finally the process was repeated a third time, with the pipette tip directed slightly laterally with respect to the first injection. Thus a total of approximately 5 μl BDA solution was injected, filling a large area of the right sensorimotor cortex. The burr hole was then covered with Gelfoam (Johnson & Johnson, Skipton, UK) and the scalp incision sutured.

Animals with dorsal column or dorsal root injuries survived for 28 days and those with CST injuries survived for 21 days. Following an anaesthetic overdose of intraperitoneal sodium pentobarbitone (Sagatal; Rhône Merieux, UK) supplemented with halothane, they were perfused transcardially with 50 ml of PBS followed by 100 ml PFA. The relevant portions of cervical, thoracic or lumbar spinal cord and dorsal roots were removed and post-fixed for 2 hours in PFA. The tissue blocks, in some cases further dissected, were then transferred to PBS containing 30% sucrose for 48 hours. The cryoprotected tissue blocks were frozen in Tissue-Tek (Sakura, Zoeterwoude, Netherlands) cooled with dry ice. Serial horizontal sections of spinal cord were cut on a freezing microtome at 40 μm and collected in 0.1 M PBS.

#### BDA immunohistochemistry

Free floating sections, maintained in serial order, were washed twice in 0.05 M TBS containing 0.5% Triton X-100 (TBST), and incubated in 0.3% H_2_O_2 _for 15 minutes, rinsed again in TBST (2 × 10 minutes) and incubated overnight in 1:200 ABC kit solution (Vector Laboratories, Burlington CA, USA) at 4°C. After two washes in TBST and one in 0.05 M TBS, sections were incubated in TSA Cy3 (1:400 in 0.05 M TBS) for 30 minutes, washed, mounted onto gelatinised slides and coverslipped with glycerol containing DABCO.

Sections were scanned with a Leica TCS NT confocal microscope. Projection images represented stacks of 10–20 optical sections merged together (2 scans at each optical section level), with a resolution of 1024 × 1024 pixels. Images from wild-type and knockout mice were acquired under identical exposure conditions.

#### CT-HRP histochemistry

Free-floating serial sections were processed to reveal CT-HRP reaction product using tetramethylbenzidine (TMB) as the chromogen, according to the method of Mesulam [51]. The sections were rinsed in dH_2_O (6 × 5 minutes), then preincubated, on ice, for 30 minutes in a solution of 0.1% sodium nitroferricyanide, 5% 0.2 M sodium acetate buffer (pH 3.3), 0.005% TMB (dissolved first in a volume of ethanol that amounted to 2.5% of the total solution) made up in dH_2_O. A solution of 0.3% H_2_O_2 _was then added (2% of the total incubation mixture volume) and the sections incubated on ice until reaction product was visible (approximately 15 minutes) at which point the reaction was arrested by 6 × 5 minute washes in 0.02 M sodium acetate buffer. Sections were collected on gelatinised slides, air dried overnight, rapidly dehydrated in ascending strength alcohol solutions and coverslipped with DPX (Merck, Poole, UK).

Digital images were taken using a Zeiss Axiophot fluorescence microscope equipped with Openlab image processing software.

### PNS injury models; functional studies

#### Sciatic nerve injury

The left sciatic nerve was exposed via a 4 mm longitudinal incision over the left mid-thigh above the level of origin of the sural nerve, and crushed for ten seconds with watchmakers' forceps (No. 7). In other mice, sciatic nerve crush was supplemented by excision of 3 mm of the saphenous nerve at mid-thigh level, in order to control for the possibility that sensory recovery following sciatic nerve injury might result from sprouting of saphenous nerve axons [52-54]. In other mice, the left sciatic nerve was transected with microscissors at the same level as the crush lesions and the cut ends immediately reanastamosed with 2 × 10/0 sutures. The skin incisions were closed with Histoacryl glue.

#### Assessment of functional recovery after sciatic (and saphenous) nerve injury

Recovery of sensory function was assessed using Von Frey hairs. Animals were placed under a glass case on a taut nylon mesh and allowed to settle for 20–30 minutes, to allow the test to be performed without restraint and under conditions of minimal stress [55]. The threshold thickness of Von Frey hair required to elicit a repeated withdrawal response was assessed in all animals, prior to nerve injury, by stimulating the lateral glabrous skin of the hindpaw. After injury, the time taken for mice to recover the ability to withdraw their paw in response to the same Von Frey hair as pre-operatively was assessed twice daily and continued for two days after recovery of function. At each testing session, the contralateral, uninjured hindpaw was assessed for comparison. Motor function was tested immediately after the sensory assessment, by assessing (recovery of) the toe spreading reflex (involuntary spreading of the digits when the hindlimbs are raised off their supporting surface by lifting the mouse by the tail).

#### Facial nerve injury and assessment of functional recovery

The right facial nerve was exposed postero-inferior to the right ear and crushed immediately distal to the stylomastoid foramen for ten seconds with watchmakers' forceps. The skin incision was closed with Histoacryl glue. Functional recovery was monitored twice daily by visually checking for recovery of spontaneous whisker twitching and for the return of the blink reflex (elicited by irritating the cornea with a thin piece of cotton wool). The left side was examined at the same time as a positive control.

### PNS injury models; anatomical studies

#### Retrograde labelling of DRG cells

After survival times of 3–88 days (Table 1), 1 μl of DiAsp was injected into the lateral plantar skin of the hindpaws both ipsilateral and contralateral to the nerve injury, under general anaesthesia, using a glass micropipette attached to a Hamilton microsyringe. Two days later, the animals were overdosed with anaesthetic and the L4 and L5 DRG on both sides removed and placed in PFA for 2 hours. The ganglia were then placed whole onto a cavity slide, coverslipped with glycerol containing DABCO, and examined and photographed immediately in a confocal microscope. If labelled cells were present, an average of 25 images was collected through the entire thickness of the DRG. Retrogradely labelled cells (visible above a fixed luminescence threshold) were counted using Openlab image processing software. The exposure time for all images was kept constant to ensure valid comparisons between control and injured and between wild-type and knockout material.

#### Silver-cholinesterase histochemistry

To examine reinnervation of muscle 7 and 10 days after sciatic nerve crush, animals were killed by overdose of anaesthetic and their right and left (control) soleus muscles removed and processed by the method of Namba *et al*. [56]. The muscles were fixed, whilst slightly stretched, in buffered formol-calcium at 4°C for 6 hours, then placed in 10% sucrose at 4°C overnight. The muscles were cut at 40 μm with a freezing microtome and the sections collected in chilled dH_2_O. Sections were then incubated for 20 minutes on ice in the following medium: acetylthiocholine (10 mg); 0.1 M sodium hydrogen maleate (13 ml); 100 mM tri-sodium citrate (1 ml); dH_2_O (2 ml); 5 mM potassium ferricyanide (2 ml); sucrose (3 g). They were then rinsed in dH_2_O and immersed in potassium ferricyanide (0.25 g/100 ml dH_2_O) for 30 seconds at room temperature and rinsed again in three washes of dH_2_O. They were then placed in absolute alcohol (2 washes over 1 hour) and returned to dH_2_O before being transferred with a glass rod to silver solution with the following composition: 50 ml dH_2_O; 0.05 g CaCO_3 _double filtered and then mixed with 0.025 g CuSO_4_.5H_2_O; 5 g AgNO_3_, for 20–30 minutes at 37°C. After rinsing for 5–20 seconds in dH_2_O the sections were placed in reducer solution (1 g hydroquinone; 10 g Na_2_SO_4_; 100 ml dH_2_O) until endplates could be visualised under a dissection microscope (usually 30–120 seconds), washed in dH_2_O, mounted on glass slides, dried at 37°C, dehydrated, passed through Histoclear (National Diagnostics, Georgia, USA) and coverslipped with DPX. Between 300 and 350 motor endplates per mouse were counted on the injured and control side and the relative proportions determined of: denervated endplates; endplates innervated by one axon; and endplates innervated by two or more axons.

#### PGP 9.5 immunohistochemistry

Reinnervation of skin was examined, after PGP 9.5 immunostaining of sensory axons, in mice surviving 17 days after sciatic nerve crush (as described above). The animals were killed by anaesthetic overdose and both hindpaws removed and placed into PFA for 3 hours, followed by immersion in 30% sucrose in TBS overnight. An area of lateral glabrous skin was dissected from each hindpaw and frozen in Tissue-Tek cooled with dry ice. Frozen sections were cut at a 12 μm perpendicular to the skin surface, collected on glass slides, air dried for 30 minutes and washed with TBS before immersion for 30 minutes in a blocking solution of 10% normal goat serum in TBS containing 0.1% Triton X-100 and 1% BSA. Sections were then reacted overnight with PGP antibody (1:500 in blocking solution) in a sealed humidified chamber at room temperature. After 3 washes in TNT, the sections were incubated with goat anti-rabbit Alexofluor 488 (1:400 in TBS) for 2 hours, followed by one rinse in TNT and 2 rinses in TBS. Slides were coverslipped using DABCO and the lateral edge of glabrous hindpaw examined and photographed (in a Zeiss Axiophot microscope using a × 20 objective lens) from a random sample of 6 sections per foot. From these images, the total numbers of axons labelled with PGP 9.5 that had regenerated into the dermis were counted in the skin samples from the ipsilateral hindpaw of all mice (Table 1), and from the contralateral hindpaw of 3 wild-type and 3 knockout mice.

#### Electron Microscopy

The extent of axonal regeneration into distal nerve branches was analysed by EM in the same group of 12 mice used to examine skin reinnervation by PGP immunohistochemistry (17 day survival) and in an additional group of mice with identical unilateral sciatic nerve crush injury and 21 day survival. Following anaesthetic overdose, the 4^th ^toe of both hind feet was amputated and placed in fixative solution (2% paraformaldehyde and 2.5% glutaraldehyde in PBS) for 24 hours followed by immersion in 5% EDTA for 48 hrs at 4°C to soften bone. The toes were post-fixed in 1% OsO_4 _in 0.1 M phosphate buffer (PB) at 4°C for 1 hour, washed in PB followed by three washes in dH_2_O, stained with 2% uranyl acetate (in dH_2_O) for 40 minutes at 4°C, washed in dH_2_O, dehydrated, passed through propylene oxide, placed in a 50:50 mixture of propylene oxide and Agar resin (12 g Agar; 8 g dodecenyl succinic anhydrite (DDSA); 5 g methylnadicanhydride; 0.4 ml benzyldimethylamine) for 45 minutes and then placed in fresh Agar resin overnight at room temperature on a rotator. Specimens were then placed in fresh resin for a further 24 hours before being placed in blocks in an oven at 60°C for 36 hours to polymerise the resin. Transverse semithin sections (ca. 1 μm) of both ipsilateral and contralateral toes were cut on an ultramicrotome and stained with toluidine blue to localise dorsal digital nerves. Ultrathin transverse sections through the nerves were cut with a diamond knife, collected on Formvar-coated slot grids, stained with lead citrate for 10–15 minutes, and examined in a JEOL 1010 electron microscope operated at 60 kV. Images were recorded on Kodak EM film 4489. In animals that had survived 17 days, numbers of unmyelinated axons in the dorsal digital nerves were counted. In the animals that survived 21 days, only remyelinating axons of all digital nerves of the 4^th ^toe were counted.

Axonal regeneration in the tibial nerve, just below the lesion site was also examined by EM in an additional group of mice, 15 days after sciatic nerve crush. These animals were given an anaesthetic overdose and perfused transcardially with 50 ml of PBS followed by 100 ml of 2% paraformaldehyde and 2.5% glutaraldehyde in PBS. A segment of tibial nerve 5 mm distal to the crush site was removed, post-fixed for 2 hours, osmicated (1 hour in 1% OsO_4 _at 4°C), dehydrated and processed into Araldite resin (10 g DDSA; 10 g Araldite (CY212); 0.8 g dibutylphthalate) warmed for two minutes at 60°C and then mixed with 0.4 ml BDMA. Following polymerisation, transverse thin sections of the tibial nerve 5 mm distal to the crush site were stained with lead citrate, examined by EM and myelinated axons counted. Independent blinded counts were made by two observers to reduce the impact of bias in relation to identification criteria for axons.

#### Retrograde labelling of facial motor neurons

The extent of facial nerve regeneration after transection was assessed with the retrograde label Fluorogold (FG). The facial nerve was transected with microscissors immediately below the stylomastoid foramen and the cut ends apposed and held together by fibrinogen. They were anaesthetised 3 days before sacrifice and a 2 × 2 mm piece of Gelfoam previously soaked with 4 μl FG (2 granules dissolved in 100 μl PBS) was placed below the skin of the right whisker pad. As a control FG was also applied to the contralateral whisker pad at the same time in most of the mice. After a further 3 days, the animals were overdosed with anaesthetic and perfused transcardially with PBS followed by PFA, as described above. A block of tissue including the pons and midbrain was dissected out and cryoprotected for 24 hours in PBS containing 30% sucrose. Frozen coronal sections through the pons (containing the facial nucleus) were cut at 40 um, collected in PBS, mounted in serial order on slides, coverslipped with DABCO, and examined immediately in a Zeiss Axiophot microscope. Counts were made of labelled neurons in the injured and control side facial nucleus (above a fixed luminescence threshold), using Openlab image processing software and the final number corrected for variability in the optical slicing of nuclei using Abercrombie's counting method [57].

In other mice, DiAsp was used as the retrograde label as a control. The facial nerve was transected and 4 μl DiAsp (2 granules dissolved in 100 μl DMSO) soaked in a Gelfoam pad placed in the whisker pad 37 days after injury, after which the animals were allowed to survive for 3 days. The facial nucleus of these animals was analysed as described above.

In addition, 6 of the 8 mice used for functional assessment of facial nerve recovery after *crush *injury were also used for anatomical assessment by retrograde labelling with DiAsp. They were anaesthetised 12 days after the facial nerve crush and DiAsp placed in the whisker pad. After a further 2 days, the animals were given an anaesthetic overdose and their facial nuclei examined as described above.

### Statistical analysis

Differences between wild-type and knockout mice with respect to both functional and anatomical analyses of axonal regeneration were evaluated for significance by using Student's two-tailed unpaired t-test when there were 6 or more animals per experimental group, or a Mann-Whitney test if there were fewer animals or if the data from any group did not represent a normal distribution. In one experiment, the standard deviation of a group was zero, which precludes such statistical analysis and a two-tailed Fisher's exact test was used to calculate a p value. For all experiments, a p value of < 0.05 was considered to be significant.

## Abbreviations

BDA, Biotinylated dextran amine; BSA, Bovine serum albumin; CNS, Central nervous system; CSPG, Chondroitin sulphate proteoglycan; CST, Corticospinal tract; CT-HRP, Cholera toxin subunit B conjugated to horseradish peroxidase; DABCO 1, 4-diazabicyclo [2,2,2] octane; DC, Dorsal column; DDSA, Dodecenyl succinic anhydrite; DiAsp, 4-Di-10-Asp; DREZ, Dorsal root entry zone; DRG, Dorsal root ganglion; EM, Electron microscopy; EDTA, Ethylenediaminetetraacetic acid; FG, Fluorogold; KO, Knockout; P, Phosphate buffer; PBS, Phosphate-buffered saline; PGP, Protein gene product; PNS, Peripheral nervous system; SD, Standard deviation; SEM, Standard error of the mean; TBS, Tris-buffered saline; WT, Wild-type

## Competing interests

The author(s) declares that there are no competing interests.

## Authors' contributions

MKHI and PNA carried out most of the work described in this paper, some of it in conjunction with KR. WBS provided the NG2 knockout mice and anti-NG2 antibody, and had input into the final paper. PNA and ARL designed the experiments. MKHI, PNA and ARL wrote the paper. All authors read and approved the final manuscript.
